# Discovery and validation of breast cancer subtypes

**DOI:** 10.1186/1471-2164-7-231

**Published:** 2006-09-11

**Authors:** Amy V Kapp, Stefanie S Jeffrey, Anita Langerød, Anne-Lise Børresen-Dale, Wonshik Han, Dong-Young Noh, Ida RK Bukholm, Monica Nicolau, Patrick O Brown, Robert Tibshirani

**Affiliations:** 1 Department of Statistics, Stanford University, Stanford, CA, USA; 2 Department of Surgery, Stanford University School of Medicine, Stanford, CA, USA; 3 Department of Genetics, Institute for Cancer Research, Rikshospitalet-Radiumhospitalet Medical Center, Oslo, Norway; 4 Medical Faculty, University of Oslo, Oslo, Norway; 5 Department of Surgery, Seoul National University College of Medicine, Seoul, Korea; 6 Department of Surgery, Akershus University Hospital, Nordbyhagen, Norway; 7 University of Oslo, Oslo, Norway; 8 Department of Biochemistry, Stanford University School of Medicine, Stanford, CA, USA; 9 Department of Health Research and Policy, Stanford University School of Medicine, Stanford, CA, USA

## Abstract

**Background:**

Previous studies demonstrated breast cancer tumor tissue samples could be classified into different subtypes based upon DNA microarray profiles. The most recent study presented evidence for the existence of five different subtypes: normal breast-like, basal, luminal A, luminal B, and ERBB2^+^.

**Results:**

Based upon the analysis of 599 microarrays (five separate cDNA microarray datasets) using a novel approach, we present evidence in support of the most consistently identifiable subtypes of breast cancer tumor tissue microarrays being: ESR1^+^/ERBB2^-^, ESR1^-^/ERBB2^-^, and ERBB2^+ ^(collectively called the ESR1/ERBB2 subtypes). We validate all three subtypes statistically and show the subtype to which a sample belongs is a significant predictor of overall survival and distant-metastasis free probability.

**Conclusion:**

As a consequence of the statistical validation procedure we have a set of centroids which can be applied to any microarray (indexed by UniGene Cluster ID) to classify it to one of the ESR1/ERBB2 subtypes. Moreover, the method used to define the ESR1/ERBB2 subtypes is not specific to the disease. The method can be used to identify subtypes in any disease for which there are at least two independent microarray datasets of disease samples.

## Background

Perou *et al*. (2000) reported evidence for breast cancer tumor subtypes defined by gene expression patterns. From the results of hierarchical clustering of 65 breast cancer and normal breast samples based on their pattern of expression of 496 *intrinsic genes*, Perou *et al*. (2000) defined four groups: basal-like, *Erb-B2 *^+^, normal-breast-like, and luminal epithelial/ER^+^. (The *intrinsic genes *were those genes whose variation was significantly greater between samples from different tumors than between samples from the same tumor before and after treatment.) The members of the basal-like group were characterized by high expression of basal keratins 5/6 and 17. The oncoprotein *ERBB2 *was relatively overexpressed in the *Erb-B2*^+ ^group members. The normal-breast-like samples resembled normal breast tissue samples: basal epithelial cell and adipose cell genes were relatively highly expressed and luminal epithelial cell genes were expressed at relatively low levels. Samples in the luminal epithelial/ER^+ ^group came from patients who were estrogen receptor (ER) positive and whose breast luminal cell markers were relatively overexpressed [1].

As new data became available these breast cancer subtypes were re-evaluated. Not only were the breast cancer subtype definitions modified, clinical outcomes were found to be significantly different between the subtypes. Sørlie *et al*. (2001) retained the basal-like, *Erb-B2*^+ ^(re-named ERBB2^+ ^in Sørlie *et al*. (2001)), and normal-breast-like groups but divided the luminal epithelial/ER^+ ^group into three subtypes: luminal A, luminal B, and luminal C. Centroids corresponding to each of these six groups were made on one dataset and used to classify the samples of another dataset. All six groups were found in the second dataset and differences in overall survival between the five groups (ERBB2^+ ^and luminal B were combined) were significant [2].

Two years later, Sørlie *et al*. (2003) re-examined 84 of the 85 arrays used in the 2001 study and added 38 new breast cancer tumor tissue arrays. Once again they found the basal-like, normal breast-like, and ERBB2^+ ^groups in their hierarchical clustering dendrogram. The number of luminal groups, however, was reduced from three to two: luminal A and luminal B. Using centroids derived from the five groups, they classified samples in two independent datasets to each of the groups. In one of the independent data sets the time to distant metastasis curves significantly differed in the five groups [3]. (A similar analysis was not carried out for the other dataset because follow-up data were not available.)

The classification of the independent datasets was also compared to the hierarchical clustering dendrograms of these datasets. In some cases, the samples classified to the same subtype grouped together in the dendrogram. This observation led to the development of an array cluster validation method described in Kapp and Tibshirani (2006). In this paper, subtype centroids are used to classify samples independent of those used to define the subtypes. The quality of the groups of samples classified to the same subtype is measured by a quantity called the *in-group proportion*. The in-group proportion is the proportion of samples in the group whose nearest-neighbor is also in the same group. Thus, a high in-group proportion indicates a group is cohesive (members of the group are close to each other). The in-group proportion uses prediction error as a measure of cluster quality. This is not a new idea, using prediction error to gauge cluster quality has appeared in several earlier studies: Dudoit and Fridlyand (2002), Dudoit, Fridlyand, and Speed (2002), and Tibshirani and Walther (2005).

To determine whether a group is significantly cohesive, the in-group proportions are compared to null distributions generated by repeatedly permuting the centroids within the box aligned with their principal components. If an actual group's in-group proportion is higher than 100(1 - *α*) of the in-group proportions which comprise the null distribution, then the corresponding subtype is validated at the *α *significance level. Using this method and the three datasets in Sørlie *et al*. (2003), the basal-like, luminal B, and ERBB2^+ ^subtypes were validated at an *α *= 0.05 significance level in at least one of the datasets. The normal breast-like and luminal A subtypes, however, were not validated at an *α *= 0.05 significance level [4]. The goal of this study was not to modify and validate previously defined subtypes. Instead, this study started from the beginning, as Perou *et al*. (2000) did, just with more microarray data. We also shared the same goal: to examine as many breast cancer tumor microarrays as possible and discover subtypes which may be biologically significant.

But our approach for identifying possible subtypes differed from previous studies. The previous studies hierarchically clustered the breast tissue samples' basal expression of a group of genes selected based on characteristics deemed *a priori *useful for classification. These selection criteria, however, do not guarantee these genes will be those which define subtypes. Since hierarchical clustering is designed to be influenced by all the data, genes which have no relationship with the subtypes but which are included in the clustering could prevent the dendrogram from partitioning the samples into the subtypes.

**Figure 1:**
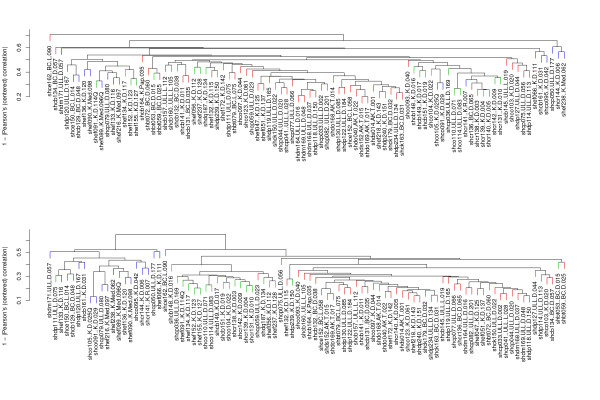
**Hierarchical clusterings of training dataset**. Hierarchical clustering of all the training dataset samples (upper) on all 23,9946 genes and (lower) on the 1,908 genes that define the three ERBB2/ESR1 subtype centroids. In both dendrograms, the training dataset samples are colored according to which ESR1/ERBB2 subtype they belong. ESR1^+^/ERBB2^- ^samples are in red; ERBB2^+ ^samples are in green; and ESR1^-^/ERBB2^- ^samples are in blue.

Figure 1 presents two dendrograms to illustrate this phenomenon. Ninety-eight samples were hierarchically clustered (average linkage) twice. In Figure 1 (upper), they were clustered using all available genes (23,946 genes); in Figure 1 (lower), they were clustered on the 1,908 genes that compose the ESR1/ERBB2 subtypes centroids. (How these subtypes are defined and how the 1,908 genes were found is described in later sections.) In both dendrograms, a sample's branch was colored according to which ESR1/ERBB2 sample group it belonged. Branches of the same color are much more cohesive in Figure 1 (lower) than in Figure 1 (upper). Therefore, the choice of the genes used to cluster the samples had a great impact upon the hierarchical dendrogram.

In general, subtypes can be thought of as being defined by a collection of genes, some of which correlate highly and some of which do not correlate highly. For samples which belong to the same subtype, the expression levels of these genes are similar; for samples which belong to different subtypes, the expression levels of these genes differ. In addition, the relationship between these genes' expression levels for all the samples in the subtype is found consistently (across datasets).

This concept of subtypes and the ideas illustrated in Figure 1 motivated our approach to identifying possible subtypes. Instead of starting with a single hierarchical clustering of the samples on a large group of biologically relevant genes, we hierarchically clustered the samples multiple times on pairs of genes that passed the test described in Step 2 (Procedure subsection of the Methods section) and so we considered likely to define subtypes. Each hierarchical cluster yielded candidate sets of subtypes which we attempted to validate. If the sample groups were validated in the training dataset, validation datasets, and demonstrated clinical differences, we examined the genes whose expression significantly differed between the groups to see if any genes correlated poorly with the genes that induced the groups.

This approach is similar to that used in the previous papers: the samples are hierarchically clustered to define candidate subtypes and then validated. Our approach is much more thorough, however. Many different sets of candidate subtypes are considered and validation is done statistically and by comparing the clinical outcomes. Moreover, except for the statistical validation procedure, all the other algorithms are standard and have been used in published microarray papers. The statistical validation procedure is new and the only option (to our knowledge) for cluster validation using an independent dataset.

Using this approach, we find three subtypes of breast cancer tissues which were validated in the training and validation datasets. In other words, they are consistently found in all datasets used in this study. We also show which of the three subtypes a sample belonged to was a significant predictor of overall survival (death irrespective of cause) and distant metastasis-free probability (first recurrence event was distant metastasis) in the validation dataset for which we had clinical data. Furthermore, we demonstrated that the subtypes were not solely based upon a local pattern of gene expression. Not all the genes which were significantly differentially expressed between the subtypes correlated highly with the pair of genes initially used to identify the subtypes.

## Results and discussion

### Results

#### Gene likely to specify subtypes

Once all the filters were applied, only 133 of the 23,946 genes in the training dataset were considered candidate genes [see Additional file 1].

#### Statistical validation of possible subtypes

As described in the Procedure subsection (Methods section), two genes that defined two sample groups which had at least one gene significantly different between the two sample groups were used to hierarchically cluster the training dataset samples. Then each dendrogram was cut to make a fixed number of sample groups (three or four). The centroids associated with the groups were derived using the nearest shrunken centroids method and used to classify the training dataset samples. When the dendrograms were cut to make three groups, 129 pairs of genes defined sample groups which all validated at the *α *= 0.05 significance level in the training dataset [see Additional file 2]. In contrast, when the dendrogram was cut to make four groups, only three pairs of genes defined sample groups which all validated at the *α *= 0.05 significance level in the training dataset (Table 1).

Of the 129 pairs of genes which resulted in three groups that were all validated in the training dataset, two pairs' candidate subtypes also were validated at the *α *0.05 significance level in all of the validation datasets. These pairs are in **bold **in Additional file 2 and their *p*-values are in Additional file 4 Tables 1, 2. These tables also show that if an *α *= 0.01 significance level had been used, no gene pair would have proceeded to Step 5 described in the Procedure subsection of the Methods section. Of the pairs of genes which resulted in four sample groups that were all validated in the training dataset, no pair's groups were validated at the *α *= 0.05 significance level in all of the validation datasets.

#### Biological validation of possible subtypes

The NKI dataset included clinical data on overall survival and on distant metastasis-free probability (DMFP), defined using distant metastasis as the first recurrence event. Using this clinical data we determined whether or not the thirteen sets of candidate subtypes which were validated in all datasets had significant clinical differences. First, the significance of the differences in the Kaplan-Meier survival and DFMP curves were tested using the Cox-Mantel log-rank test. Second, the likelihood ratio test was used to determine whether or not the classifications were significant factors in predicting overall survival or DMFP in the presence of other common risk factors.

For both sets of groups, the log-rank test of the differences between the survival and DMFP curves were significant at the *α *= 0.05 level (Table 2). In addition, the groups induced by *BCMP11 *(UniGene Cluster ID Hs.100686) and *ABCC11 *(UniGene Cluster ID Hs.335891) were significant predictors of overall survival and DMFP when other known risk factors were included in the model at the *α *= 0.05 and *α *= 0.10 levels, respectively. Moreover, the groups induced by *SLG39A6 *(UniGene Cluster ID Hs.79136) and *GATA3 *(UniGene Cluster ID Hs.524134) were significant predictors of overall survival (but not of DMFP) at the *α *= 0.10 significance level (Table 2).

**Figure 2:**
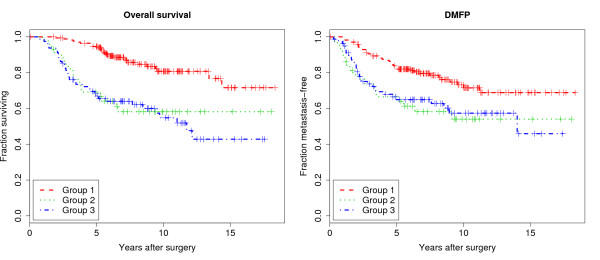
**Kaplan-Meier curves for overall survival and DMFP for the three groups defined by BCMP11/ABCC11**. The Kaplan-Meier survival curves (left) and DMFP curves (right) for each of the three groups defined by *BCMP11 *and *ABCC11*.

For the groups defined by *BCMP11 *and *ABCC11*, Groups 2 and 3 were associated with worse overall survival and worse DMFP when compared to Group 1 (Table 3). Also, belonging to Group 2 or Group 3 was a significant (*α *= 0.05) predictor for overall survival in the presence of other known risk factors. Belonging to Group 2 was a significant (*α *= 0.10) predictor for DMFP in the presence of other known risk factors. Finally, the Kaplan-Meier curves in Figure 2 show that Group 2 and Group 3 have similar overall survival and metastasis-free probability over the short-term. After 15 years, however, Group 3 has worse overall survival and DMFP.

**Figure 3:**
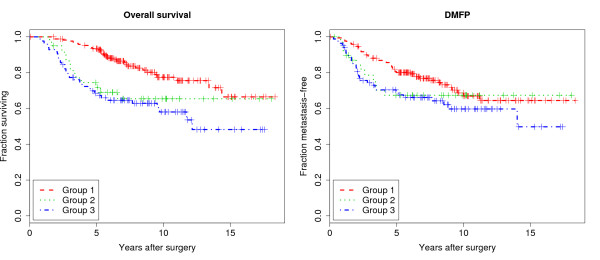
**Kaplan-Meier curves for overall survival and DMFP for the three groups defined by SLC39A6/GATA3**. The Kaplan-Meier survival curves (left) and DMFP curves (right) for each of the three groups defined by *SLC39A6 *and *GATA3*.

For the groups defined by *SLC39A6 *and *GATA3*, Group 2 and Groups 3 were associated with worse overall survival and worse DMFP when compared to Group 1 (Table 4). Only belonging to Group 3 was a significant (*α *= 0.05) predictor of overall survival in the presence of other known risk factors. The Kaplan-Meier curves in Figure 3 show that Group 3 consistently had the worse overall survival and DMFP. Group 2 initially resembled Group 3 in terms of overall survival and DMFP, but after time, Group 2 looks more like Group 1.

Notice that estrogen receptor status was not included in the multivariate analysis. Estrogen receptor status was not included because the expression of *ESR1 *has been shown to be positively correlated with the expression of *BCMP11 *and *GATA3 *[5,6]. Estrogen receptor status, expression of *BCMP11*, and expression of *GATA3 *are confounding variables. Therefore, including both estrogen receptor status and the subtypes defined by *BCMP11/ABCC11 *or *SLC39A6/GATA3 *may produce inaccurate results.

## Discussion

### *BCMP11*/*ABCC11 *and *SLC39A6*/*GATA3 *groups as subtypes

Both sets of three groups defined by the *BCMP11/ABCC11 *gene pair and the *SLCS9A6/GATAS *gene pair have many of the characteristics of subtypes (see Background section). First, they were reproducible: in both cases, the three groups were found in all the datasets at the *α *= 0.05 level [see Additional file 4 Tables 1, 2]. Second, both sets of three groups were significantly different clinically (this applies to a greater extent to the *BCMP11/ABCC11 *groups than to the *SLC39A6/GATAS*). Based upon the log-rank test the survival differences and DMFP differences between the three *BCMP11/ABCC11 *groups and between the three *SLC39A6/GATAS *groups were significant at the *α *= 0.05 level (Table 2 and Figures 2, 3). Furthermore, at the *α *= 0.10 level, the *BCMP11/ABCC11 *groups were significant predictors of overall survival and distant metastasis as first recurrence event. At the same level of significance, the *SLC39A6/GATAS *groups were significant predictors of overall survival, but not of distant metastasis as first recurrence event (Tables 3, 4).

**Figure 4:**
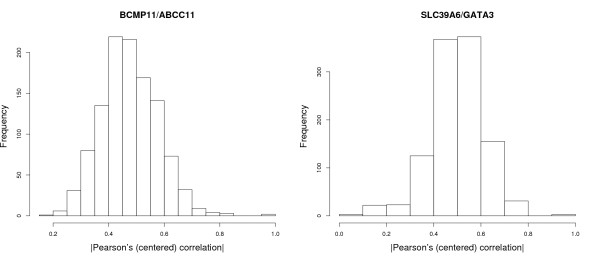
**Histograms of correlations between significantly differentially expressed genes and the genes that induced them**. (Left) Histogram of the maximum absolute Pearson's (centered) correlation of the genes that are significantly differentially expressed between the *BCMP11/ABCC11 *groups with *BCMP11 *and with *ABCC11*. (Right) Histogram of the maximum absolute Pearson's (centered) correlation of the genes that are significantly differentially expressed between the *GATA3/SLC39A6 *groups with *GATA3 *and with *SLC39A6*.

Figure 4 demonstrates that both sets of three groups also possess the final desirable characteristic of subtypes as defined in the Background section: Genes which do not correlate highly with the genes used to define the centroids are significantly differentially expressed between the groups. For the *BCMP11/ABCC11 *groups 1,121 genes were significantly differentially expressed between the three groups (determined by SAM, Δ = 0.49315 and the 90th percentile of false positives = 0). The absolute Pearson's (centered) correlation was computed between each gene and *BCMP11*, and between each gene and *ABCC11*. Figure 4 (left) is the histogram of the maximum of those two values for all 1,121 genes. Similarly, for the *SLC39A6/GATAS *groups 1,103 genes were significantly differentially expressed between the three groups (Δ = 0.47040 and the 90th percentile of false positives = 0). Figure 4 (right) is the histogram of the maximum of the correlations between *SLC39A6 *and each of the 1,103 genes and between *GATAS *and each of the 1,103 genes.

In both histograms, some of the area of the histogram is below 0.5. Therefore, for both sets of three groups, genes which significantly differed between the three groups were not always highly (positively or negatively) correlated with the genes which defined the groups.

### Similarity of *BCMP11*/*ABCC11 *and *SLC39A6*/*GATA3 *groups

Of the 1,121 genes which significantly differed between the *BCMP11/ABCC11 *groups, 78.06% were also significantly differentially expressed between the *SLC39A6/GATAS *groups. Eight-hundred and seventy-five genes were significantly differentially expressed between the two sets of groups. In fact, all 204 genes used to define the *SLC39A6/GATA3 *centroids were included in the 1,908 genes used to define the *BCMP11/ABCC11 *centroids. Furthermore, when the classifications of the training dataset were compared, all of the members of *SLC39A6/GATA3 *Group 1 were in *BCMP11/ABCC11 *Group 1, all of the members of *SLC39A6/GATA3 *Group 3 were in *BCMP11/ABCC11 *Group 3, and the majority of the members of *SLC39A6/GATA3 *Group 2 were in the *BCMP11/ABCC11 *Group 2 (Table 5 upper).

Therefore, the groups defined by the two sets of centroids are very similar. Since the *BCMP11/ABCC11 *groups were more significantly different clinically, we will restrict our attention to them.

**Figure 5:**
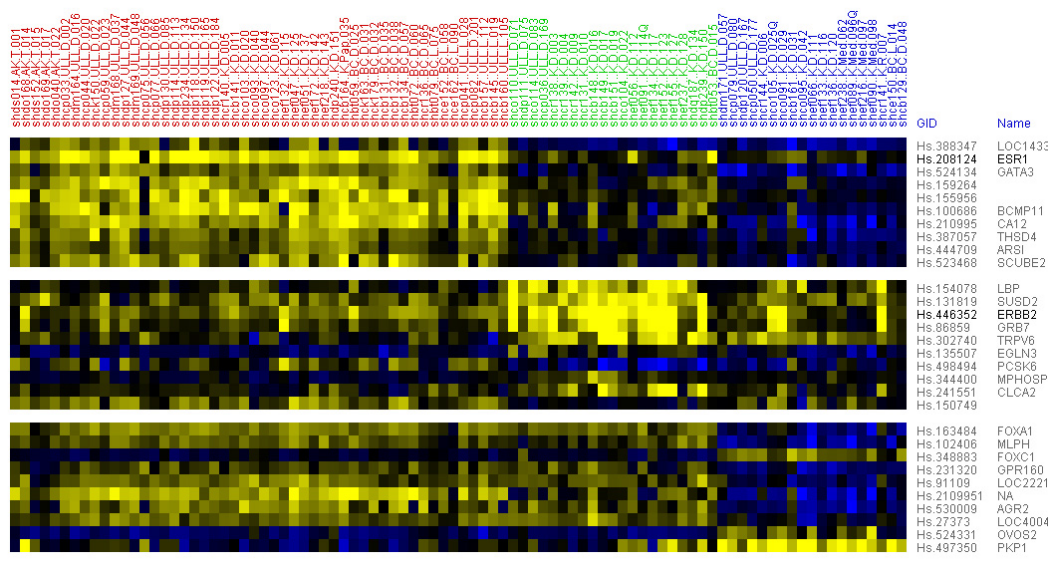
**Training dataset samples dendrogram clustered on BCMP11/ABCC11 centroid genes**. Thirty centroid genes present in all datasets that best distinguish the three *BCMP11/ABCC11 *groups. The first group of genes are the top ten genes that distinguish Group 1 from Groups 2 and 3; the second group of genes are the top ten genes that distinguish Group 2 from Groups 1 and 3; and the last group of genes are the top ten genes that distinguish Group 3 from Groups 1 and 2. The samples in *BCMP11/ABCC11 *Group 1 (ERBB2^-^/ESR1^+^) are in red; the samples in *BCMP11/ABCC11 *Group 2 (ERBB2^+^) are in green; and the samples in the *BCMP11/ABCC11 *Group 3 (ESR1^-^/ERBB2^-^) are in blue.

For each *BCMP11/ABCC11 *subtype, the genes most significantly differentially expressed between it and the other two *BCMP11/ABCC11 *were found using SAM and the training dataset. In Figure 5, the most significantly differentially expressed genes present in all datasets and used to define the *BCMP11/ABCC11 *centroids for each SAM analysis are shown.

Notice that the expression of the estrogen receptor 1 (*ESR1*) distinguishes Group 1 from Groups 2 and 3. *ESR1 *is overexpressed in Group 1 and underexpressed in Groups 2 and 3. Moreover, *ERBB2 *is overexpressed in Group 2, but underexpressed in Groups 1 and 3. As a consequence, from this point on, *BCMP11/ABCC11 *Group 1 will be referred to as the ESR1^+^/ERBB2^- ^subtype; *BCMP11/ABCC11 *Group 2 will be referred to as the ERBB2+ subtype; and *BCMP11/ABCC11 *Group 3 will be referred to as the ESR1-/ERBB2- subtype. Collectively, they will now be referred to as the ESR1/ERBB2 subtypes.

### Comparison of ESR1/ERBB2 subtypes and Sørlie et al. (2003) subtypes

One of the ESR1/ERBB2 subtypes' names is the same as one of the Sørlie *et al*. (2003) subtypes: ERBB2^+^. Although none of the ESR1/ERBB2 subtypes exactly matched any of the Sørlie *et al*. (2003) subtypes, the vast majority of the samples in four of the Sørlie *et al*. (2003) subtypes fell in one ESR1/ERBB2 subtype (Table 5 lower). First, all of the normal-like samples, all of the luminal A samples, and 9 of the eleven luminal B samples were classified to the ESR1^+^/ERBB2^- ^subtype. Second, 7 of the 11 samples classified to the Sørlie *et al*. (2003) ERBB2^+ ^subtype were also classified to the ESR1/ERBB2 ERBB2^+ ^subtype. Finally, the samples classified to the basal subtype were fairly evenly distributed among the three ESR1/ERBB2 subtypes.

### Existence of other subtypes

Even though we only have shown evidence for the existence of three subtypes, that does not preclude the existence of other subtypes. For example, using fluorescent in situ hybridization (FISH) and histological staining, some breast cancer tumors belong to ERBB2 amplified and ER+ group [7]. Therefore, one may have expected this study to have found an ESR1^+^/ERBB2^+ ^subtype. In our training and testing datasets, the few patients who we knew were ERBB2 amplified based upon FISH did not have especially high *ESR1 *expression levels. So the most likely explanation for not finding an ERBB2^+^/ESR1^+ ^subtype is that ERBB2 and ER amplified samples did not form a group distinct from the ERBB2 amplified but ER unamplified samples. Nevertheless, three of the four samples known to be ESR1^+^/ERBB2^+ ^by FISH were classified to the ERBB2^+ ^group. The fourth sample was classified to the ESR1^+^/ERBB2^- ^group.

## Conclusion

To summarize, we started from the very beginning without consideration for the results described in Perou *et al*. (2000), Sørlie *et al*. (2001), or Sørlie *et al*. (2003). With microarray data from hundreds of breast tissue samples (normal and tumor), we compiled a collection of 133 candidate genes based upon percentage of data present, variation, and ability to create two groups of samples significantly differing in a large number of genes. Using that collection of candidate genes, pairs of genes which generated three or four candidate sample subtypes that were found in the training and validation datasets were identified. Of these two sets of three groups, only one set had candidate subtypes which were significant predictors (*α *= 0.10) of overall survival and DMFP (when other known risk factors, not including estrogen receptor status, were controlled for): the ESR1/ERBB2 subtypes. Thus we have identified three breast cancer tumor subtypes that are consistently identified in all the datasets we examined and have biological relevance.

This paper is intended to be a starting point for further research. The ESR1/ERBB2 centroids can be applied to any microarray sample indexed by UniGene Cluster ID to classify it to one of the three subtypes. Thus, these centroids represent a standard way of determining the subtype of a breast cancer tumor tissue sample. As a consequence, further research is needed to determine whether or not sub-populations of these subtypes exist. If they do, clinical and biological characterization of them will be required.

Although this paper focused on breast cancer, it also provides an answer to this question: How does one identify subtypes? Just as our subtypes differ from those in previous papers, our answer to this question also differs. Instead of using of all of the genes likely to define subtypes to hierarchically cluster the training dataset samples, we only used pairs of genes in our hierarchical clusterings to prevent genes that are independent of a sample partition from obscuring it (see Figure 1). This approach proved to be fruitful and produced two similar sets of groups which were found in all our datasets and were significant predictors of overall survival or DMFP. Therefore, the methods presented here are useful for identifying subtypes of any disease provided that at least two independent microarray datasets of diseased samples are available.

## Methods

### Materials

Five microarray datasets were used in this study (three of which have appeared in previously published papers): NKI dataset [8,9], Sørlie dataset [3], Zhao/Langerød dataset [10], Korean dataset and Norway/Stanford dataset. [See Additional file 3 for detailed lists of the arrays in each dataset.] The Sørlie and Zhao/Langerød datasets are publicly available from the Stanford Microarray Database (SMD) [11]. When data were downloaded from SMD, the control spots were included but the flagged spots were not. All of the arrays associated with Zhao *et al*. (2004) were used, but only the arrays not used for selection of the intrinsic genes (*disease_state_design*) from the Sørlie *et al*. (2003) paper were used.

The NKI dataset and associated clinical data are also publicly available [12]. Before this file was used, however, the UniGene Cluster IDs were updated (Build 180 released January 20, 2005) and the logarithm base was changed from ten to two to make it compatible with the other datasets.

The Norway/Stanford dataset was composed of samples from 60 tumors, 45 of which came from Ullevål University Hospital (Norway) and 15 of which came from Akershus University Hospital (Norway). DNA microarray analysis was carried out at Stanford University Medical Center (United States of America), however. The 81 arrays in the Korean dataset were from patients at Seoul National University College of Medicine.

For the Norway/Stanford, Korean, Zhao, and NKI datasets, but not the Sørlie dataset, the RNA was amplified prior to hybridization to the cDNA microarrays [13,14].

The validation method used in this paper required a training dataset and at least one independent testing dataset. The Norway/Stanford, Korean, and Zhao/Langerød datasets were all made on the same microarray platform using the same RNA preparation protocol and were indexed by clone ID (not UniGene cluster ID), so they could be easily combined and used as training and testing datasets. Tumor samples were taken from three different populations and the distributions of the samples' clinical characteristics in the Norway/Stanford, Korean, and Zhao/Langerød datasets are not equal, however, so we followed the procedure described below and divided each of the three datasets and subsequently pooled groups to make the training dataset and the testing dataset. If we had used the Norway/Stanford and Korean datasets as the training dataset and the Zhao/Langerød dataset as the test set (or some similar combination), we risked finding subtypes that were specific to a group of subjects.

**Figure 6:**
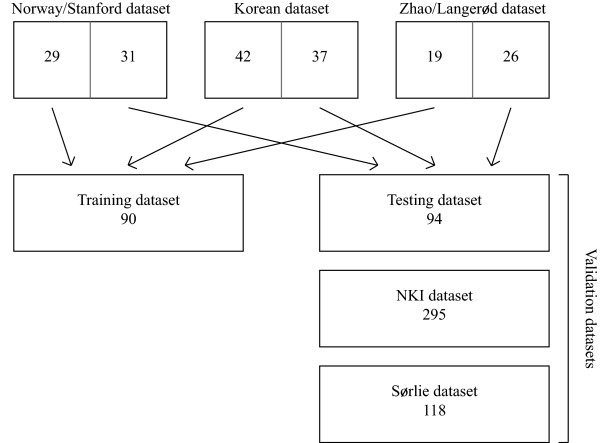
**Training and testing datasets formation**. This diagram shows how the training dataset and testing dataset were formed. For the top row, the numbers in the boxes represent the number of samples that were combined to form the training dataset and testing dataset. The arrows point to the dataset in which they were put.

More specifically, each of the Norway/Stanford, Korean, and Zhao/Langerød datasets was randomly divided into a training set and testing set of approximately equal sizes. Next, the three training sets were combined and the three testing sets were combined. The arrays from normal patients and redundant arrays (arrays which appeared in more than one dataset) were subsequently removed leaving 90 arrays in the training dataset and 96 arrays in the testing dataset. The Sørlie and NKI datasets were used for validation. The testing dataset, Sørlie dataset, and NKI dataset taken together are referred to as the *validation datasets *(Figure 6).

The random splits of the Norway/Stanford, Korean, and Zhao/Langerød datasets were done once. As long as the composition of the training and testing datasets are similar, the results should not depend upon these random splits. More specifically, if one subtype is absent from the training or testing dataset, the results will be affected. As will be shown in the Statistical analysis subsection below, the three breast cancer subtypes we identified are found in the training dataset and all the validation datasets.

Furthermore, an examination of Additional file 4 Figure 1 shows that the proportions of samples in each of the three subtypes are high enough to make it unlikely that all the samples of one or more subtypes will be absent from the training dataset or testing dataset after a random split.

Finally, before they were used, the training, testing, and Sørlie datasets' annotation was changed from clone ID to UniGene Cluster ID. In each of the three datasets, if a clone corresponded to more than one UniGene Cluster ID, then the clone's entries were replicated. The entries for clones which corresponded to the same UniGene Cluster ID were then averaged together over the clones. The correspondence between UniGene Cluster ID and clone ID was dictated by *UniGene_Curated_110504*, a curated synthetic gene list provided by SMD. This gene list does not include defective clones (*e.g*. incorrectly labeled clones).

### Statistical analysis

The public domain statistical program R version 2.2.0 [15,16] was used for all analysis. In addition to many functions in the base R package, the Significance Analysis of Microarrays (SAM) [17] function in the R package *siggenes *and the Prediction Analysis for Microarrays (PAM) R package *pamr *were used to form centroids and analyze the sample groups. PAM implements the nearest shrunken centroids method described in Tibshirani *et al*. (2002).

Statistical validation was done according to the procedure described in Kapp and Tibshirani (2006) (an implementation of the algorithm is in the R package *clusterRepro*) while biological validation was done using the *coxph *(fits Cox proportional hazards models) and *survdiff *(applies the Cox-Mantel log-rank test) functions in the R *survival *package.

### Procedure

The procedure we followed was more complicated than that of the previous papers. The additional complexity was a reasonable price to pay for a more comprehensive search for the subtypes of breast cancer, however. Instead of *a priori *choosing a single set of subtype-defining genes (*e.g*. an intrinsic gene list), as was done in the earlier papers, we defined a subset of genes we considered likely to define subtypes based on the test described in Step 2 and used them to generate 17,556 (= 2 × (1332)
 MathType@MTEF@5@5@+=feaafiart1ev1aaatCvAUfKttLearuWrP9MDH5MBPbIqV92AaeXatLxBI9gBaebbnrfifHhDYfgasaacH8akY=wiFfYdH8Gipec8Eeeu0xXdbba9frFj0=OqFfea0dXdd9vqai=hGuQ8kuc9pgc9s8qqaq=dirpe0xb9q8qiLsFr0=vr0=vr0dc8meaabaqaciaacaGaaeqabaqabeGadaaakeaadaqadiqaauaabeqaceaaaeaacqaIXaqmcqaIZaWmcqaIZaWmaeaacqaIYaGmaaaacaGLOaGaayzkaaaaaa@320E@) sets of possible subtypes. Then we examined every set seeking the ones that could be biologically and statistically validated. Our procedure is described in detail below.

#### Step 1. Filter genes

**Figure 7:**
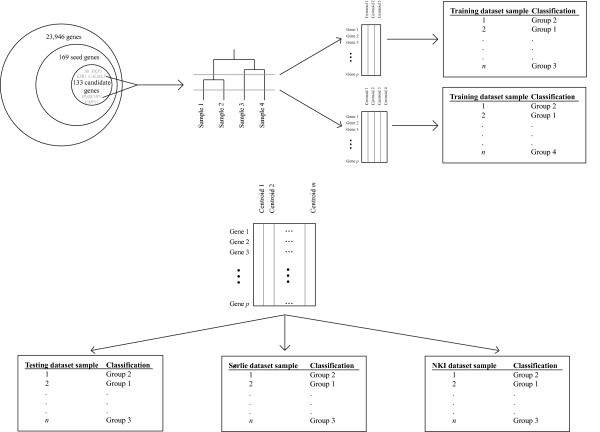
**Steps of the procedure**. Pictorial representation of steps 1 – 5 described in the Procedure subsection of the Methods section. (Upper) Filter all 23,946 genes by removing genes with at least 10% missing data or a standard deviation less than 1.5. Keep all seed genes that define two training dataset sample groups between which at least one of the 23,946 genes is significantly differentially expressed. Repeatedly do the following steps. Select two of the 133 candidate genes and hierarchically cluster the training dataset sample on these two genes. Cut the dendrogram from the top down to produce three groups of samples. Cut the same dendrogram from the top down again to produce four groups of samples. Use PAM to determine which of the 23,946 genes best define centroids for the training dataset sample groups obtained from the dendrogram. Form the centroids by taking only the data for those genes and averaging over the sample classified to the same group. Use the centroids to classify the training dataset samples. (Lower) If all the groups are validated in the training dataset then use the centroids to classify the testing datasets' samples. If all the groups are validated in all of the validation datasets, then the significance of the groups' clinical difference is determined (not pictured).

Initially each of the training dataset genes (indexed by UniGene Cluster ID) was considered for determining subtypes. Genes which had a standard deviation less than 1.5 and had less than 90% of data present in the training data were removed. The percentage of genes which had a standard deviation less than 1.5 was 98.6%. Of those 327 genes, 169 had at least 90% data present (196 genes had at least 85% genes present and 229 genes had at least 80% data present). Therefore, the number of genes was reduced from 23,946 to 169 (Figure 7).

After this step, the missing data in all four datasets were imputed with the gene average. In each dataset, the missing values for a given gene were replaced by the average of the present expression values for that gene. From this point on, none of the datasets contains any missing values.

#### Step 2. Identify genes likely to specify subtypes

Each of the 169 seed genes which passed through the filters was used individually to hierarchically cluster (Euclidean distance, average linkage) the training dataset samples (R function *hclust*). The R function *cutree *automatically cut each dendrogram (from the top down) to form two groups of samples. The two-sample unpaired *T*-statistic was used to determine the number of genes whose expression levels differed significantly between the two groups. (These *T*-statistics were not related to the in-group proportion or the validation of subtypes.) For a given sample grouping and each gene *j *(1 ≤ *j *≤ 23,946), the following statistic was computed:

Tj=Xj¯−Yj¯Sj1nj+1mj     (1)
 MathType@MTEF@5@5@+=feaafiart1ev1aaatCvAUfKttLearuWrP9MDH5MBPbIqV92AaeXatLxBI9gBaebbnrfifHhDYfgasaacH8akY=wiFfYdH8Gipec8Eeeu0xXdbba9frFj0=OqFfea0dXdd9vqai=hGuQ8kuc9pgc9s8qqaq=dirpe0xb9q8qiLsFr0=vr0=vr0dc8meaabaqaciaacaGaaeqabaqabeGadaaakeaacqWGubavdaWgaaWcbaGaemOAaOgabeaakiabg2da9maalaaabaWaa0aaaeaacqWGybawdaWgaaWcbaGaemOAaOgabeaaaaGccqGHsisldaqdaaqaaiabdMfaznaaBaaaleaacqWGQbGAaeqaaaaaaOqaaiabdofatnaaBaaaleaacqWGQbGAaeqaaOWaaOaaaeaadaWcaaqaaiabigdaXaqaaiabd6gaUnaaBaaaleaacqWGQbGAaeqaaaaakiabgUcaRmaalaaabaGaeGymaedabaGaemyBa02aaSbaaSqaaiabdQgaQbqabaaaaaqabaaaaOGaaCzcaiaaxMaadaqadiqaaiabigdaXaGaayjkaiaawMcaaaaa@4690@

where

Sj2=∑i=1nj(Xi,j−Xj¯)2+∑i=1mj(Yi,j−Yj¯)2nj+mj−2.     (2)
 MathType@MTEF@5@5@+=feaafiart1ev1aaatCvAUfKttLearuWrP9MDH5MBPbIqV92AaeXatLxBI9gBaebbnrfifHhDYfgasaacH8akY=wiFfYdH8Gipec8Eeeu0xXdbba9frFj0=OqFfea0dXdd9vqai=hGuQ8kuc9pgc9s8qqaq=dirpe0xb9q8qiLsFr0=vr0=vr0dc8meaabaqaciaacaGaaeqabaqabeGadaaakeaacqWGtbWudaqhaaWcbaGaemOAaOgabaGaeGOmaidaaOGaeyypa0ZaaSaaaeaadaaeWaqaamaabmGabaGaemiwaG1aaSbaaSqaaiabdMgaPjabcYcaSiabdQgaQbqabaGccqGHsisldaqdaaqaaiabdIfaynaaBaaaleaacqWGQbGAaeqaaaaaaOGaayjkaiaawMcaamaaCaaaleqabaGaeGOmaidaaOGaey4kaSYaaabmaeaadaqadiqaaiabdMfaznaaBaaaleaacqWGPbqAcqGGSaalcqWGQbGAaeqaaOGaeyOeI0Yaa0aaaeaacqWGzbqwdaWgaaWcbaGaemOAaOgabeaaaaaakiaawIcacaGLPaaaaSqaaiabdMgaPjabg2da9iabigdaXaqaaiabd2gaTnaaBaaameaacqWGQbGAaeqaaaqdcqGHris5aaWcbaGaemyAaKMaeyypa0JaeGymaedabaGaemOBa42aaSbaaWqaaiabdQgaQbqabaaaniabggHiLdGcdaahaaWcbeqaaiabikdaYaaaaOqaaiabd6gaUnaaBaaaleaacqWGQbGAaeqaaOGaey4kaSIaemyBa02aaSbaaSqaaiabdQgaQbqabaGccqGHsislcqaIYaGmaaGaeiOla4IaaCzcaiaaxMaadaqadiqaaiabikdaYaGaayjkaiaawMcaaaaa@6747@

The *X*_*i,j *_were the expression levels for gene *j *for the samples in the first group defined by the dendrogram and the *Y*_*i,j *_were the expression levels for the same gene for the samples in the second group defined by the dendrogram. There were *n*_*j *_data for gene *j *in the first group and *m*_*j *_data for gene *j *in the second group. The genes which defined groups with at least one *T*_*j *_significant at the *α *= 0.05 level were considered genes likely to define subtypes (called candidate genes). Seed genes that induced two sample groups between which a lot of genes were significantly differentially expressed were not treated any differently than seed genes that induced two sample groups between which one gene was significantly differentially expressed: both became candidate genes. Of the 169 seed genes, 133 were candidate genes: genes that defined two groups between which at least one gene was significantly differentially expressed. (See Figure 7.)

This is a very simple way to look for differentially expressed genes. A more sophisticated approach is described in McLachlan *et al*. (2002). They identify differentially expressed genes by fitting a mixture of two densities (Gaussian or Student's *t*). The likelihood ratio test is used to determine whether or not two densities fit better than a single density. If that is so, a gene is considered to be differentially expressed. This method for filtering genes is implemented in the first step of the EMMIX-GENE algorithm [18]. An even more sophisticated approach to the identification of differentially expressed genes can be found in McLachlan *et al*. (2006). When we used the former procedure to filter genes, none of the 169 seed genes were considered to be significantly differentially expressed (results not shown).

#### Step 3. Combine genes pairwise to identify possible subtypes

Each of the 133 candidate genes was combined with every other candidate gene to generate candidate sets of subtypes. Each pair of candidate genes was used to hierarchically cluster (Euclidean distance, average linkage) the training data samples. A pair of genes, instead of more, was used because it was the most simple choice and least likely to miss obvious, broad groupings of samples. If real subtypes were defined by two genes, the inclusion of one or more additional genes may have prevented the hierarchical dendrogram from identifying the real subtypes. Single genes were not used because that would essentially replicate the previous step.

All of the 8,778 (= (1332)
 MathType@MTEF@5@5@+=feaafiart1ev1aaatCvAUfKttLearuWrP9MDH5MBPbIqV92AaeXatLxBI9gBaebbnrfifHhDYfgasaacH8akY=wiFfYdH8Gipec8Eeeu0xXdbba9frFj0=OqFfea0dXdd9vqai=hGuQ8kuc9pgc9s8qqaq=dirpe0xb9q8qiLsFr0=vr0=vr0dc8meaabaqaciaacaGaaeqabaqabeGadaaakeaadaqadiqaauaabeqaceaaaeaacqaIXaqmcqaIZaWmcqaIZaWmaeaacqaIYaGmaaaacaGLOaGaayzkaaaaaa@320E@) dendrograms were cut twice: first to define three groups and then to define four groups. A gene that is differentially expressed between groups of samples will be differentially expressed between at least two sample groups. Therefore, when two such genes are combined to partition the samples, at the very least they will partition the samples into two groups (*e.g*., the genes are highly correlated), three groups (*e.g*., one gene subdivides one of the two sample groups induced by the other gene), or four groups (*e.g*., both genes divide the samples into two independent sets of two groups). When a pair of genes defines two sample groups they are most likely very similar to at least one set of the two sample groups defined by the individual genes. We have already chosen the candidate genes to divide the samples into two potentially interesting groups. The next logical step is to look for additional complexity in the interaction between pairs of genes. Therefore, the hierarchical dendrogram was cut to make three groups and four groups.

For each of the two cuts, the nearest shrunken centroids method identified the fewest number of genes (out of 23,946 genes) needed to form centroids minimizing the cross-validation misclassification error. Since the nearest shrunken centroids method used cross-validation, if any of the three (or four) groups had fewer than five samples, centroids corresponding to the classification were not made and no attempt to validate the groups was made. In addition, if there were fewer than three genes in the centroids, validation of the associated groups was not attempted because the Pearson's (centered) correlation coefficient between samples could not be computed without at least three genes. Once the nearest shrunken centroids method identified the genes, a set of (three or four) centroids was made by averaging these genes' expression levels over the samples classified to the same candidate subtype. (See Figure 7.)

As in Step 2, alternative methods for executing Step 3 exist. In the EMMIX-GENE procedure of McLachlan *et al*. (2006), for example, a large collection of genes is reduced to a smaller representative set. All of the genes are clustered and a few representative members of the groups stand in for the entire group. If this method was used, the computation could be greatly reduced and a larger gene list could have been considered. Using this method, however, requires one to select the representative member of each group. The correct way to do that is not obvious.

#### Step 4. Attempt to statistically validate the possible subtypes

Each candidate set of centroids formed in the previous step was applied to classify the training dataset in an attempt to validate the corresponding possible subtypes. If all of the possible subtypes were validated at the *α *= 0.05 significance level, then the possible subtypes were pursued in all the validation datasets. (See Figure 7.)

#### Step 5. Analyze the clinical differences between the possible subtypes

Each candidate set of centroids corresponding to possible subtypes validated in the training dataset and all the validation datasets were used to classify the NKI samples because survival and distant metastasis data were available for this dataset.

The likelihood ratio and the Cox-Mantel log-rank tests were used to determine whether or not the survival or distant metastasis-free probability (DMFP) differences between the possible subtypes were significant. (See Figure 7.)

#### Overall level of significance

Throughout this analysis an *α *= 0.05 level of significance was used. The level of significance for the entire procedure may not be *α *= 0.05, however. At four distinct junctures in the analysis, *α *played a role but was not adjusted to account for multiple hypothesis testing. First, *α *= 0.05 was used to determine which of the 169 seed genes were candidate genes. Second, *α *= 0.05 was used to determine which gene pairs induced sets of groups that were validated in the training dataset. Third, *α *= 0.05 was used to determine which of the sets of groups that were validated in the training dataset were also validated in the testing datasets. Finally, *α *= 0.05 was used to determine which of sets of groups that were validated in all datasets were different clinically.

In the first three steps of the analysis many Type I errors could be generated, but they are very unlikely to pass through the latter two steps. To estimate the rate of false positives, a cursory analysis was carried out (data not shown). The data in each row of all the datasets were permuted independently. Then the analysis described above to was conducted for a portion of the sets of three groups that were generated. Of the 100 sets examined, thirty-two were validated in the training dataset, but none were validated in all of the datasets. This suggest that while early steps in the procedure are prone to Type I errors, the later steps reduce the number of Type I errors to a level below *α *= 0.05.

## Authors' contributions

AVK performed all the analyses and wrote the manuscript. Several different microarray datasets were used. SSJ and ALBD were involved in the designing and running of the Norway/Stanford microarray experiments. IRKB collected the Akershus tumor tissue samples (the AK Norway/Stanford experiments in Additional file 3), and AL performed some of the Norway/Stanford microarray experiments. For the Korean dataset, SSJ, D-YN, and WH designed and supervised the microarray experiments, and D-YN and WH collected the tumor tissue samples. SSJ, MN, FOB, and RT all read the manuscript and made significant revisions to the manuscript. In addition, RT made significant contributions to the statistical analysis. All authors have read and approved the final manuscript.

## Supplementary Material

Additional File 1 This is a multi-page table that lists the 133 candidate genes. Each of these 133 candidate genes induced two groups which had at least one gene significantly (*α *= 0.05) differentially expressed between the two sample groups. Click here for file

Additional File 2 This is the multi-page table that lists the pairs of genes resulting in three groups all validated in the training dataset at the *α *= 0.05 significance level. Click here for file

Additional File 4 This file contains the supporting information for the manuscript. Scatterplots of the training data and tables of *p*-values from the validation procedure for the *BCMP11/ABCC11 *groups and the *GATA3/SLC39A6 *groups are presented. Click here for file

Additional File 3 This contains the accession information necessary to obtain the microarray data from the Stanford Microarray Database. Click here for file

## Figures and Tables

**Table 1:** Pairs of genes resulting in four groups all validated in the training dataset at the *α *= 0.05 significance level.

**First gene**	**Second gene (s)**
Hs.512643	*GRB7*
Hs.1657	*LTF*
*ESR1*	*PDZK1IP1*

The pairs of genes which induced four sample groups which all validated in the training dataset (*α *= 0.05) are listed. The first column lists the first gene in the pairs and the second column lists the second genes in the pairs. The UniGene Cluster ID is provided when the gene symbol is unavailable.

**Table 2:** Significance of overall survival and DFMP differences for groups validated in all datasets.

Gene pair	Survival log-rank test *p*-value	DMFP log-rank test *p*-value	Survival likelihood ratio test *p*-value	DMFP likelihood ratio test *p*-value
*BCMP11/ABCC11*	6.7188 × 10^-8^	**0.0005464543**	**0.005674599**	*0.06732856*
*SLC39A6/GATA3*	4.836347 × 10^-5^	**0.03727548**	*0.0516708*	0.4392585

The results of the Cox-Mantel log-rank test and the likelihood ratio test for the four sets of groups which were statistically validated in all datasets are presented. The significance of the differences between the three groups in each set in overall survival and DMFP were tested. The *p*-values between 0.10-0.05 are in *italics *and *p*-values less than 0.05 are in bold.

**Table 3:** Multivariate analysis of mortality and DMFP risk factors and BCMP11/ABCC11.

	Overall survival		Distant metastasis-free probability	
	
	Hazard ratio (95% confidence interval)	*p*-value	Hazard ratio (95% confidence interval)	*p*-value
*BCMP11/ABCC11*: Group 2 vs. Group 1	2.124 (1.0923 – 4.130)	**0.0260**	1.759 (0.948 – 3.263)	*0.073*
*BCMP11/ABCC11*: Group 3 vs. Group 1	2.155 (1.1874 – 3.913)	**0.0120**	1.452 (0.817 – 2.580)	0.200
Age (decade)	0.688 (0.4792 – 0.988)	**0.0430**	0.736 (0.523 – 1.035)	*0.078*
Tumor diameter (cm)	1.022 (0.9967 – 1.048)	*0.0890*	1.034 (1.010 – 1.058)	**0.006**
Lymph node status (positive node)	1.010 (0.8309 – 1.228)	0.9200	1.045 (0.886 – 1.234)	0.600
Tumor grade: Poorly diff. vs. Intermediate	1.185 (0.6586 – 2.131)	0.5700	0.979 (0.560 – 1.713)	0.940
Tumor grade: Well diff. vs. Intermediate	0.240 (0.0828 – 0.698)	**0.0088**	0.403 (0.190 – 0.855)	**0.018**
Vascular invasion: 1–3 vessels vs. ≥ 4 vessels	0.627 (0.2150 – 1.829)	0.3900	0.530 (0.198 – 1.424)	0.210
Vascular invasion: 0 vessels vs. ≥ 4 vessels	0.589 (0.1322 – 2.621)	0.4900	0.560 (0.145 – 2.158)	0.400
Mastectomy vs. breast conserving therapy	1.119 (0.6933 – 1.807)	0.6400	1.193 (0.750 – 1.895)	0.460
Chemo and hormonal therapy v. no adjuvant therapy	0.724 (0.3281 – 0.1596)	0.4200	0.502 (0.246 – 1.022)	*0.057*

Results of the multivariate analysis of mortality risk factors (left) and DMFP risk factors (right). The group classifications determined by the *BCMP11 *and *ABCC11 *gene pair are included. The *p*-values less than 0.05 are in bold. The *p*-values significant at an *α *= 0.10 level but not at an *α *= 0.05 significance level are in *italics*.

**Table 4:** Multivariate analysis of mortality and DMFP risk factors and SLC39A6/GATA3.

	Overall survival		Distant metastasis-free probability	
	
	Hazard ratio (95% confidence interval)	*p*-value	Hazard ratio (95% confidence interval)	*p*-value
*SLC39A6/GATA3*: Group 2 vs. Group 1	1.557 (0.7914 – 3.061)	0.2000	1.208 (0.621 – 2.347)	0.5800
*SLC39A6/GATA3*: Group 3 vs. Group 1	1.675 (0.9739 – 2.882)	*0.0620*	1.207 (0.715 – 2.038)	0.4800
Age (decade)	0.678 (0.4722 – 0.973)	**0.0350**	0.720 (0.513 – 1.011)	*0.0580*
Tumor diameter (cm)	1.022 (0.9964 – 1.048)	*0.0940*	1.033 (1.009 – 1.058)	**0.0067**
Lymph node status (positive node)	1.019 (0.8396 – 1.237)	0.8500	1.054 (0.894 – 1.241)	0.5300
Tumor grade: Poorly diff. vs. Intermediate	1.403 (0.7994 – 2.463)	0.2400	1.117 (0.657 – 1.901)	0.6800
Tumor grade: Well diff. vs. Intermediate	0.222 (0.0769 – 0.643)	**0.0055**	0.381 (0.180 – 0.806)	**0.0120**
Vascular invasion: 1–3 vessels vs. ≥ 4 vessels	0.678 (0.2352 – 1.956)	0.4700	0.577 (0.217 – 1.532)	0.2700
Vascular invasion: 0 vessels vs. ≥ 4 vessels	0.638 (0.1456 – 2.794)	0.5500	0.597 (0.157 – 2.263)	0.4500
Mastectomy vs. breast conserving therapy	1.078 (0.6682 – 1.738)	0.7600	1.165 (0.733 – 1.851)	0.5200
Chemo and hormonal therapy v. no adjuvant therapy	0.718 (0.3291 – 1.567)	0.4100	0.496 (0.245 – 1.005)	*0.0510*

Results of the multivariate analysis of mortality risk factors (left) and DMFP risk factors (right). The group classifications determined by the *SLC39A6 *and *GATA3 *(Hs.524134) gene pair are included. The *p*-values less than 0.05 are in bold. The *p*-values significant at an *α *= 0.10 level but not at an *α *= 0.05 significance level are in *italics*.

**Table 5:** Comparison with BCMP11/ABCC11 subtypes.

		*BCMP11/ABCC11*		
		Group 1	Group 2	Group 3
*SLC39A6/GATA3*	Group 1	42	0	6
	Group 2	8	21	3
	Group 3	0	0	16

Sørlie subtype	Normal-like	6	0	0
	ERBB2^+^	4	7	0
	Luminal A	28	0	0
	Luminal B	9	2	0
	Basal	5	8	6

(Upper) Each of the training dataset samples was classified twice: first to one of the *SLC39A6/GATA3 *groups and then to one of the *BCMP11/ABCC11 *subtypes. The entries in the table are the number of samples classified to the *SLC39A6/GATA3 *(row) subtype and to the *BCMP11/ABCC11 *subtype (column). (Lower) A subset of the Norway/Stanford samples were classified to one of the three subtypes presented in this paper and the classification is compared with the Sørlie *et al*. (2003) classification. Each entry in the table is the number of Norway/Stanford samples classified to the Sørlie *et al*. (2003) subtype (row) and the *BCMP11/ABCC11 *subtype (column)
