# Discovery and validation of breast cancer subtypes

**DOI:** 10.1186/1471-2164-8-101

**Published:** 2007-04-13

**Authors:** Amy V Kapp, Stefanie S Jeffrey, Anita Langerød, Anne-Lise Børresen-Dale, Wonshik Han, Dong-Young Noh, Ida RK Bukholm, Monica Nicolau, Patrick O Brown, Robert Tibshirani

**Affiliations:** 1 Department of Statistics, Stanford University, Stanford, CA, USA; 2 Department of Surgery, Stanford University School of Medicine, Stanford, CA, USA; 3 Department of Genetics, Institute for Cancer Research, Rikshospitalet-Radiumhospitalet Medical Center, Oslo, Norway; 4 Medical Faculty, University of Oslo, Oslo, Norway; 5 Department of Surgery, Seoul National University College of Medicine, Seoul, Korea; 6 Department of Surgery, Akershus University Hospital, Nordbyhagen, Norway; 7 University of Oslo, Oslo, Norway; 8 Department of Biochemistry, Stanford University School of Medicine, Stanford, CA, USA; 9 Department of Health Research and Policy, Stanford University School of Medicine, Stanford, CA, USA

## Abstract

Following the publication of our recent article (Kapp *et al.*, *BMC Genomics 2006*, 7:231), we (the authors) regrettably found several errors in the published Table 5. This correction article not only describes what makes the published Table 5 incorrect, it also presents the correct Table 5.

I regret to say that several errors exist in Table 5 of our recently published paper [1]. Here we present the correct version of Table 5 and explain what is incorrect about the published Table 5. (See Table 1 of this manuscript.)

A typographical error exists in Table 5 (upper panel). The 6 that is presented in the upper right-hand corner of the published Table 5 (upper panel) should be a zero. The correct version of Table 5 (upper panel) is shown in Table 2 of the present manuscript.

The paragraphs in the paper associated with Table 5 (upper panel) are correct as published.

Table 5 (lower panel) has a more significant error. The correct version of Table 5 (lower panel) is shown in Table 3 of the present manuscript.

What was presented in the paper was a comparison of the Sørlie *et al. *(2003) classifications of the Sørlie dataset (without a cutoff), not a subset of the Norway/Stanford arrays.

Moreover, the ***Comparison of ESR1/ERBB2 subtypes and Sørlie *et al. *(2003) subtypes ***section is incorrect. The text in this section is associated with Table 5 (lower panel) and describes the results for the Sørlie dataset, not the results for a subset of the Norway/Stanford dataset as intended (and as stated in the table's legend). Based upon the correct Table 5 (lower panel) which presents the results for the Norway/Stanford arrays of samples from Ullevål University Hospital, the subtypes described by [2] are fairly similar to the ESR1/ERBB2 subtypes we defined. All of the luminal A and luminal B samples were classified to Group 1 (the ESR1^+^/ERBB2^- ^subtype). All but one of the Ullevål samples in the Norway/Stanford dataset that were classified to the *ERBB2*-overexpressing Sørlie *et al. *(2003) subtype were classified by us to Group 2 (the ERBB2^+ ^subtype). Finally, except for one sample, all of the basal samples were classified to Group 3 (the ESR1^-^/ERBB2^- ^subtype). All of the samples that were not classified to any of the Sørlie *et al. *(2003) subtypes were classified to our ESR1^+^/ERBB2^- ^subtype rather than being uniformly distributed among all three of our subtypes.

It may be surprising to see one of the basal samples in our ERBB2^+^subtype and not with the rest of the basal samples in our ESR1^-^/ERBB2^- ^subtype. In all other papers, the basal subtype has been the most cohesive. Our ESR1/ERBB2 centroids consist of approximately four times as many genes as the Sørlie *et al. *(2003) centroids do (1908 genes and 496 genes, respectively). Not all of the genes in the ESR1/ERBB2 centroids belong to the ESR1 gene cluster or to the ERBB2 gene cluster. It is the influence of the genes which do not belong to these clusters that causes one of the basal samples to not be classified with the others.

Table 4 of the present document contains the 45 arrays used to make the correct Table 5 (lower panel). Anita Langerød classified each array to one of the Sørlie *et al. *(2003) subtypes if the array's correlation with one of the subtypes centroids was at least 0.2. These classifications will appear in an upcoming publication.

## Figures and Tables

**Table 1:** The (original) incorrect Table 5 as published in Kapp *et al. *(2006).

		*BCMP11*/*ABCC11*		
		Group 1	Group 2	Group 3
*SLC39A6*/*GATA3*	Group 1	42	0	6
	Group 2	8	21	3
	Group 3	0	0	16

Sørlie subtype	Normal-like	6	0	0
	ERBB2^+^	4	7	0
	Luminal A	28	0	0
	Luminal B	9	2	0
	Basal	5	8	6

**Table 2:** The correct Table 5 (upper panel).

		*BCMP11/ABCC11*		
		Group 1	Group 2	Group 3
*SLC39A6/GATA3*	Group 1	42	0	0
	Group 2	8	21	3
	Group 3	0	0	16

**Table 3:** The correct Table 5 (lower panel).

		*BCMP11/ABCC11*		
		Group 1	Group 2	Group 3
Sørlie subtype	ERBB2+	1	6	0
	Luminal A	15	0	0
	Luminal B	3	0	0
	Basal	0	1	8
	None	11	0	0

**Table 4:** The arrays used to make the correct Table 5 (lower panel)

**Array**	**Sørlie *et al. *(2003) subtype**	**ESR1/ERBB2 subtype**
ULL-D-002	Luminal A	ESR1^+^/ERBB2^-^
ULL-D-016	Luminal A	ESR1^+^/ERBB2^-^
ULL-D-020	Luminal A	ESR1^+^/ERBB2^-^
ULL-D-022	Luminal A	ESR1^+^/ERBB2^-^
ULL-D-023	Luminal B	ESR1^+^/ERBB2^-^
ULL-D-037	Luminal A	ESR1^+^/ERBB2^-^
ULL-D-044	Luminal A	ESR1^+^/ERBB2^-^
ULL-D-048	Luminal A	ESR1^+^/ERBB2^-^
ULL-D-056	Luminal B	ESR1^+^/ERBB2^-^
ULL-D-057	Basal-like	ESR1^-^/ERBB2^-^
ULL-D-066	None	ESR1^+^/ERBB2^-^
ULL-D-071	ERBB2	ERBB2^+^
ULL-D-075	Basal-like	ERBB2^+^
ULL-D-080	Basal-like	ESR1^-^/ERBB2^-^
ULL-D-083	ERBB2	ERBB2^+^
ULL-D-085	None	ESR1^+^/ERBB2^-^
ULL-D-113	Luminal B	ESR1^+^/ERBB2^-^
ULL-D-134	Luminal A	ESR1^+^/ERBB2^-^
ULL-D-150	Luminal A	ESR1^+^/ERBB2^-^
ULL-D-165	None	ESR1^+^/ERBB2^-^
ULL-D-167	Basal-like	ESR1^-^/ERBB2^-^
ULL-D-169	ERBB2	ERBB2^+^
ULL-D-177	Basal-like	ESR1^-^/ERBB2^-^
ULL-D-184	None	ESR1^+^/ERBB2^-^
ULL-D-007	None	ESR1^+^/ERBB2^-^
ULL-D-011	Luminal A	ESR1^+^/ERBB2^-^
ULL-D-013	Luminal A	ESR1^+^/ERBB2^-^
ULL-D-026	Basal-like	ESR1^-^/ERBB2^-^
ULL-D-027	ERBB2	ERBB2^+^
ULL-D-038	None	ESR1^+^/ERBB2^-^
ULL-D-053	None	ESR1^+^/ERBB2^-^
ULL-D-065	Basal-like	ESR1^-^/ERBB2^-^
ULL-D-067	None	ESR1^+^/ERBB2^-^
ULL-D-074	Luminal A	ESR1^+^/ERBB2^-^
ULL-D-087	ERBB2	ESR1^+^/ERBB2^-^
ULL-D-096	ERBB2	ERBB2^+^
ULL-D-099	Basal-like	ESR1^-^/ERBB2^-^
ULL-D-101	ERBB2	ERBB2^+^
ULL-D-122	Luminal A	ESR1^+^/ERBB2^-^
ULL-D-132	None	ESR1^+^/ERBB2^-^
ULL-D-135	None	ESR1^+^/ERBB2^-^
ULL-D-139	Basal-like	ESR1^-^/ERBB2^-^
ULL-D-143	Luminal A	ESR1^+^/ERBB2^-^
ULL-D-144	Luminal A	ESR1^+^/ERBB2^-^
ULL-D-183	None	ESR1^+^/ERBB2^-^
