# Supplementary material for: Discovery and validation of breast cancer subtypes
Source: BMC Genomics. 2006 Sep 11;7:231. doi: 10.1186/1471-2164-7-231 (PMC1574316; doi:10.1186/1471-2164-7-231)
Supplement: Additional File 1 — This is a multi-page table that lists the 133 candidate genes. Each of these 133 candidate genes induced two groups which had at least one gene significantly (α = 0.05) differentially expressed between the two sample groups. [file 1471-2164-7-231-S1.pdf]

Candidate gene list - Each of these 133 candidate genes induced two groups which had at least one gene significantly ($\alpha = 0.05$) differentially expressed between the two sample groups.

**UniGene Cluster ID**	**Gene Symbol**	**Gene Name**	**Number of significant genes**
Hs.512126		Data not found	9527
Hs.438683	*S8*	SLAM family member 8	9255
Hs.512643		MRNA; cDNA DKFZp762C115 (from clone DKFZp762C115)	9091
Hs.1657		Data not found	9072
Hs.208124	*ESR1*	Estrogen receptor 1	9072
Hs.64568		Transcribed locus, moderately similar to XP_234686.3 PREDICTED: similar to immunoglobulin heavy chain [Rattus norvegicus]	8884
Hs.100686	*UMP11*	Breast cancer membrane protein 11	8825
Hs.431099	*PDZK1IP1*	PDZK1 interacting protein 1	8218
Hs.497350	*PKP1*	Plakophilin 1 (ectodermal dysplasia/skin fragility syndrome)	8126
Hs.131740		Transcribed locus, strongly similar to XP_520662.1 PREDICTED: similar to male sterility domain containing 1; cDNA sequence BC055759 [Pan troglodytes]	8064
Hs.180142	*CALML5*	Calmodulin-like 5	7960
Hs.388347	*LOC143381*	Hypothetical protein LOC143381	7797
Hs.100431	*CXCL13*	Chemokine (C-X-C motif) ligand 13 (B-cell chemoattractant)	7789
Hs.109425		Data not found	7680
Hs.444372		Data not found	7680
Hs.7413		Transcribed locus	7457
Hs.79136	*SLC39A6*	Solute carrier family 39 (zinc transporter), member 6	7444

Candidate gene list - Each of these 133 candidate genes induced two groups which had at least one gene significantly ($\alpha = 0.05$) differentially expressed between the two sample groups.

Hs.155956		Transcribed locus, strongly similar to NP_000653.3 N-acetyltransferase 1; arylamide acetylase 1 (N-acetyltransferase 1); arylamine N-acetyltransferase-1 [Homo sapiens]	7319
Hs.458430		Data not found	7319
Hs.405944		Data not found	7268
Hs.449585	*IGL@*	Immunoglobulin lambda locus	7268
Hs.376984	*SOX10*	SRY (sex determining region Y)-box 10	7214
Hs.1955	*SAA2*	Serum amyloid A2	7192
Hs.416854		Data not found	7187
Hs.458275		Data not found	7085
Hs.199487	*RERG*	OAS-like, estrogen-regulated, growth inhibitor	7046
Hs.155223		Data not found	7006
Hs.233160	*STC2*	Stanniocalcin 2	7006
Hs.332649		Transcribed locus, strongly similar to XP_498081.1 PREDICTED: similar to Olfactory receptor 2I2 [Homo sapiens]	6959
Hs.525874		Data not found	6948
Hs.210995	*CA12*	Carbonic anhydrase XII	6767
Hs.279916		Data not found	6767
Hs.391828		Transcribed locus, strongly similar to XP_030559.1 PREDICTED: PAR-6 beta [Homo sapiens]	6688
Hs.406050	*DNALI1*	Dynein, axonemal, light intermediate polypeptide 1	6654
Hs.97220	*CHAD*	Chondroadherin	6647
Hs.504115	*TRIM29*	Tripartite motif-containing 29	6536
Hs.333303	*GJB1*	Gap junction protein, beta 1, 32kDa (connexin 32, Charcot-Marie-Tooth neuropathy, X-linked)	6488

Candidate gene list - Each of these 133 candidate genes induced two groups which had at least one gene significantly ($\alpha = 0.05$) differentially expressed between the two sample groups.

Hs.29190	*C1orf64*	Chromosome 1 open reading frame 64	6432
Hs.2256	*MMP7*	Matrix metallopeptidase 7 (matrilysin, uterine)	6394
Hs.58076	*GRETAL10-11*	Keratin associated protein 10-11	6359
Hs.154078	*LBP*	Lipopolysaccharide binding protein	6325
Hs.16530		Data not found	6303
Hs.26225	*GABBI*	Gamma-aminobutyric acid (GABA) A receptor, pi	6243
Hs.268573	*GSTT1*	Glutathione S-transferase theta 1	6170
Hs.432677		Data not found	6137
Hs.524438		Data not found	6137
Hs.7644	*HIST1H1C*	Histone 1, H1c	6098
Hs.41690	*DSC3*	Desmocollin 3	6069
Hs.77367	*CXCL11*	Chemokine (C-X-C motif) ligand 11	6048
Hs.439760	*CYP4X1*	Cytochrome P450, family 4, subfamily X, polypeptide 1	6027
Hs.150821		Data not found	6006
Hs.38972	*TSPAN1*	Tetraspanin 1	5994
Hs.789	*CXCL1*	Chemokine (C-X-C motif) ligand 1 (melanoma growth stimulating activity, alpha)	5989
Hs.75643	*NFE2*	Nuclear factor (erythroid-derived 2), 45kDa	5974
Hs.8944	*PCOLCE2*	Procollagen C-endopeptidase enhancer 2	5942
Hs.103253	*PLIN*	Perilipin	5929
Hs.524293		Full-length cDNA clone CS0DI085YD17 of Placenta Cot 25-normalized of Homo sapiens (human)	5918
Hs.458446		Immunoglobulin variable region germ-line transcripts (RF.BM, YF.PB2 cell line)	5912
Hs.446352	*ERBB2*	V-erb-b2 erythroblastic leukemia viral oncogene homolog 2, neuro/glioblastoma derived oncogene homolog (avian)	5882

Candidate gene list - Each of these 133 candidate genes induced two groups which had at least one gene significantly ($\alpha = 0.05$) differentially expressed between the two sample groups.

Hs.1925	*DSG3*	Desmoglein 3 (pemphigus vulgaris antigen)	5874
Hs.86859	*GRB7*	Growth factor receptor-bound protein 7	5871
Hs.1051	*GZMB*	Granzyme B (granzyme 2, cytotoxic T-lymphocyte-associated serine esterase 1)	5842
Hs.93194	*APOA1*	Apolipoprotein A-I	5812
Hs.169266		Data not found	5780
Hs.519057	*NPY1R*	Neuropeptide Y receptor Y1	5780
Hs.55279	*SERPINB5*	Serpin peptidase inhibitor, clade B (ovalbumin), member 5	5757
Hs.24395		Data not found	5717
Hs.95972	*SILV*	Silver homolog (mouse)	5664
Hs.382827		Data not found	5652
Hs.515966		Transcribed locus, strongly similar to XP_379089.1 PREDICTED: hypothetical protein XP_379089 [Homo sapiens]	5566
Hs.522555	*APOD*	Apolipoprotein D	5529
Hs.75736		Data not found	5529
Hs.529517	*LTF*	Lactotransferrin	5500
Hs.32405		MRNA; cDNA DKFZp586G0321 (from clone DKFZp586G0321)	5471
Hs.69771	*CFB*	Complement factor B	5413
Hs.388547		Data not found	5412
Hs.534293	*SERPINA3*	Serpin peptidase inhibitor, clade A (alpha-1 antiproteinase, antitrypsin), member 3	5412
Hs.151135	*FN3K*	Fructosamine 3 kinase	5395
Hs.182385	*HPN*	Hepsin (transmembrane protease, serine 1)	5358
Hs.69517	*LY6K*	Lymphocyte antigen 6 complex, locus K	5272
Hs.50002	*CCL19*	Chemokine (C-C motif) ligand 19	5174
Hs.99949	*PIP*	Prolactin-induced protein	5172
Hs.97774		Transcribed locus	5155

Candidate gene list - Each of these 133 candidate genes induced two groups which had at least one gene significantly ($\alpha = 0.05$) differentially expressed between the two sample groups.

Hs.229128		Transcribed locus, weakly similar to XP_209041.2 PREDICTED: similar to KIAA1503 protein [Homo sapiens]	5079
Hs.473695		Transcribed locus, strongly similar to NP_444507.1 chloride intracellular channel 6; chloride channel form A [Homo sapiens]	4960
Hs.169946		Data not found	4959
Hs.524134	*GATA3*	GATA binding protein 3	4959
Hs.101174	*MAPT*	Microtubule-associated protein tau	4952
Hs.59889	*HMGCS2*	3-hydroxy-3-methylglutaryl-Coenzyme A synthase 2 (mitochondrial)	4901
Hs.36563		Transcribed locus	4835
Hs.54451		Data not found	4819
Hs.530509		Data not found	4819
Hs.441113	*MAGEA6*	Melanoma antigen family A, 6	4798
Hs.82961	*TFF3*	Trefoil factor 3 (intestinal)	4605
Hs.430324		Data not found	4605
Hs.102406	*MLPH*	Melanophilin	4595
Hs.111676		Data not found	4464
Hs.400095	*HSPB8*	Heat shock 22kDa protein 8	4464
Hs.170195		Data not found	4334
Hs.143961	*CCL18*	Chemokine (C-C motif) ligand 18 (pulmonary and activation-regulated)	4248
Hs.239600	*CALML3*	Calmodulin-like 3	4196
Hs.26770	*FABP7*	Fatty acid binding protein 7, brain	4194
Hs.459642	*CACNA1H*	Calcium channel, voltage-dependent, alpha 1H subunit	4134
Hs.3041	*UNG2*	Uracil-DNA glycosylase 2	4117
Hs.302740	*TRPV6*	Transient receptor potential cation channel, subfamily V, member 6	4115
Hs.84905	*KRT20*	Keratin 20	4097

Candidate gene list - Each of these 133 candidate genes induced two groups which had at least one gene significantly ($\alpha = 0.05$) differentially expressed between the two sample groups.

Hs.534221	*ADAMTS15*	ADAM metallopeptidase with thrombospondin type 1 motif, 15	4070
Hs.80342		Data not found	4066
Hs.334131		Data not found	4050
Hs.464829	*CDH2*	Cadherin 2, type 1, N-cadherin (neuronal)	4050
Hs.117874		Data not found	3926
Hs.498494	*PCSK6*	Proprotein convertase subtilisin/kexin type 6	3926
Hs.9029	*KRT23*	Keratin 23 (histone deacetylase inducible)	3766
Hs.505326	*NELL2*	NEL-like 2 (chicken)	3692
Hs.413924		Data not found	3604
Hs.154138		Data not found	3544
Hs.234642	*AQP3*	Aquaporin 3 (Gill blood group)	3456
Hs.335891	*ABCC11*	ATP-binding cassette, sub-family C (CFTR/MRP), member 11	3442
Hs.525647		Data not found	3357
Hs.17518	*RSAD2*	Radical S-adenosyl methionine domain containing 2	3355
Hs.375600		Data not found	3315
Hs.442180	*CILP*	Cartilage intermediate layer protein, nucleotide pyrophosphohydrolase	3216
Hs.270833	*AREG*	Amphiregulin (schwannoma-derived growth factor)	3199
Hs.272499	*DHRS2*	Dehydrogenase/reductase (SDR family) member 2	3188
Hs.176588	*CYP4Z1*	Cytochrome P450, family 4, subfamily Z, polypeptide 1	3080
Hs.251754		Data not found	3039
Hs.332053		Data not found	2980
Hs.436643	*SYT13*	Synaptotagmin XIII	2822
Hs.449587		Data not found	2725
Hs.46732	*MAOB*	Monoamine oxidase B	2650
Hs.22111	*IGSF1*	Immunoglobulin superfamily, member 1	2643

Candidate gene list - Each of these 133 candidate genes induced two groups which had at least one gene significantly ($\alpha = 0.05$) differentially expressed between the two sample groups.

Hs.411881	*GRB14*	Growth factor receptor-bound protein 14	2166
Hs.10319	*UGT2B7*	UDP glucuronosyltransferase 2 family, polypeptide B7	2054
