# Supplementary material for: Discovery and validation of breast cancer subtypes
Source: BMC Genomics. 2006 Sep 11;7:231. doi: 10.1186/1471-2164-7-231 (PMC1574316; doi:10.1186/1471-2164-7-231)
Supplement: Additional File 2 — This is the multi-page table that lists the pairs of genes resulting in three groups all validated in the training dataset at the α = 0.05 significance level. [file 1471-2164-7-231-S2.pdf]

Pairs of genes resulting in three groups all validated in the training dataset at the $\alpha = 0.05$ significance level - The pairs of genes which induced three sample groups which all validated in the training dataset ($\alpha = 0.05$) are listed. The first column lists the first gene in the pairs and the second column lists the second genes in the pairs. The UniGene Cluster ID is provided when the gene symbol is unavailable. The pairs of genes in **bold** defined three sample groups all of which were validated at the $\alpha = 0.05$ significance level in all three validation datasets.

**First gene**	**Second gene(s)**
Hs.512126	Hs.432677, *HIST1H1C*
Hs.512643	*CXCL13*, *HPN*
Hs.1657	Hs.64568, *PDZK1IP1*, *SOX10*, Hs.332649, *KRTAP10-11*, Hs.432677, Hs.524293, *GRB7*, *ABCC11*
ESR1	Hs.64568, *PDZK1IP1*, *KRTAP10-11*, Hs.524293, *GRB7*, *ABCC11*, Hs.375600
Hs.64568	Hs.432677, *SERPINB5*, Hs.530509
**BCMP11**	*SOX10*, *KRTAP10-11*, *PIP*, **ABCC11**
PDZK1IP1	*SOX10*, *SAA2*, Hs.54451, Hs.530509
PKP1	*SLC39A6*, *KRTAP10-11*, *LBP*
CALML5	*KRTAP10-11*
CXCL13	*CXCL1*
Hs.109425	*SOX10*, *STC2*
Hs.444372	*CHAD*, *APOD*, *CYP4Z1*
Hs.7413	Hs.155223, *STC2*, Hs.524293, Hs.251754
**SLC39A6**	Hs.473695, Hs.169946, **GATA3**
Hs.155956	*GSTT1*
Hs.458430	Hs.332649, *GSTT1*
Hs.405944	*SERPINB5*, *SILV*
IGL@	*SERPINB5*
SOX10	Hs.416854, Hs.155223, *STC2*, Hs.391828,*C1orf64*, *LBP*, *GRB7*, *TRPV6*, Hs.117874, *PCSK6*
SAA2	*MAOB*
Hs.416854	*ERBB2*, *PIP*

Pairs of genes resulting in three groups all validated in the training dataset at the $\alpha = 0.05$ significance level - The pairs of genes which induced three sample groups which all validated in the training dataset ($\alpha = 0.05$) are listed. The first column lists the first gene in the pairs and the second column lists the second genes in the pairs. The UniGene Cluster ID is provided when the gene symbol is unavailable. The pairs of genes in **bold** defined three sample groups all of which were validated at the $\alpha = 0.05$ significance level in all three validation datasets.

Hs.458275	*KRT23*
RERG	*GRB7*
Hs.525874	*APOD*, Hs.75736
CA12	Hs.391828
Hs.279916	Hs.391828, *LBP*
DNALI1	*ERBB2*, *GRB7*, *LTF*
CHAD	*HIST1H1C*, *TSPAN1*
TRIM29	*LBP*, *KRT20*
C1orf64	*ERBB2*
KRTAP10-11	*ABCC11*
LBP	*TSPAN1*, Hs.524293, *SERPINB5*
Hs.432677	*PLIN*, *ERBB2*, *GRB7*
Hs.524438	*ERBB2*, *PCSK6*
HIST1H1C	*ERBB2*
CYP4X1	*TFF3*
TSPAN1	*SERPINA3*, *HMGCS2*
PLIN	*GRB7*, *SERPINB5*, *TFF3*, Hs.430324
Hs.524293	*HMGCS2*
ERBB2	*LTF*, *GATA3*, Hs.54451, Hs.530509, Hs.449587
GRB7	Hs.515966, *GATA3*, Hs.36563
SERPINB5	Hs.525647
Hs.24395	*FN3K*
SILV	Hs.515966
LTF	Hs.97774
FN3K	*AREG*

Pairs of genes resulting in three groups all validated in the training dataset at the $\alpha = 0.05$ significance level - The pairs of genes which induced three sample groups which all validated in the training dataset ($\alpha = 0.05$) are listed. The first column lists the first gene in the pairs and the second column lists the second genes in the pairs. The UniGene Cluster ID is provided when the gene symbol is unavailable. The pairs of genes in **bold** defined three sample groups all of which were validated at the $\alpha = 0.05$ significance level in all three validation datasets.

HPN	*AREG*
CCL19	Hs.430324
PIP	*CACNA1H*
Hs.97774	*ABCC11*
MAPT	*ABCC11*
Hs.54451	*DHRS2*
Hs.530509	*DHRS2*
TFF3	*PCSK6*
Hs.430324	Hs.413924
CACNA1H	*NELL2*, Hs.375600, Hs.332053
PCSK6	*IGSF1*
ABCC11	Hs.525647, *MAOB*
AREG	*GRB14*
