# Supplementary material for: Discovery and validation of breast cancer subtypes
Source: BMC Genomics. 2006 Sep 11;7:231. doi: 10.1186/1471-2164-7-231 (PMC1574316; doi:10.1186/1471-2164-7-231)
Supplement: Additional File 4 — This file contains the supporting information for the manuscript. Scatterplots of the training data and tables of p-values from the validation procedure for the BCMP11/ABCC11 groups and the GATA3/SLC39A6 groups are presented. [file 1471-2164-7-231-S4.pdf]

Supporting Information

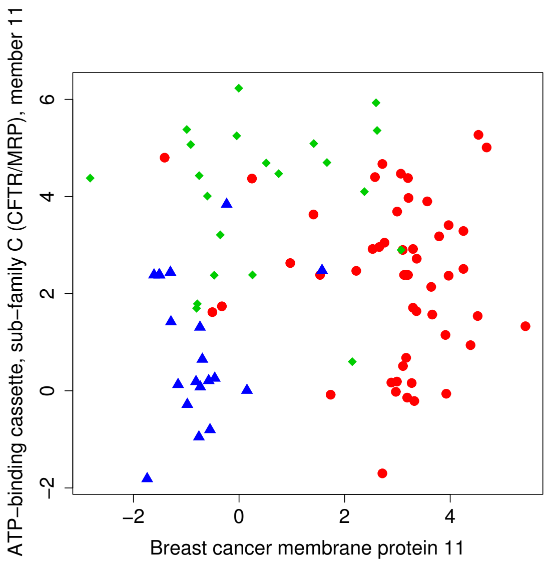

Figure 1: The expression level of *ABCC11* is plotted against the expression level of *BCMP11* for the training dataset samples. The samples are colored based upon the classification done in Step 4 of the procedure. Group 1 samples are red circles; Group 2 samples are green diamons; and Group 3 samples are blue triangles.

BCMP11/ABCC11				
Dataset	Centroid size	Group 1 p-value	Group 2 p-value	Group 3 p-value
Training dataset	1908	0.003921569 (255)	0.012165450 (411)	0 (446)
Testing dataset	1908	0.02094241 (191)	0.04130435 (460)	0 (450)
Sørlie dataset	1908	0.01840491 (163)	0 (325)	0 (645)
NKI dataset	1253	0 (84)	0 (264)	0 (262)

Table 1: The p-values for the three groups defined by *BCMP11* and *ABCC11*. The first column lists the datasets in which the three groups were validated at the $\alpha = 0.05$ level. The second column lists the number of genes in the centroids used to classify the dataset to the left. The third through fifth columns list the p-values for each of the three groups.

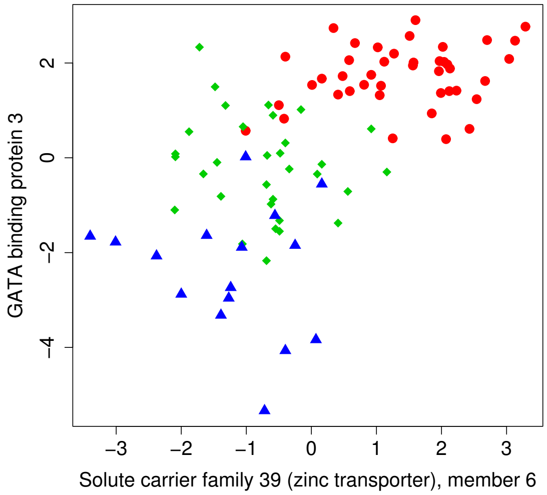

Figure 2: The expression level of *GATA3* is plotted against the expression level of *SLC39A6* for the training dataset samples. The samples are colored based upon the classification done in Step 4 of the procedure. Group 1 samples are red circles; Group 2 samples are green diamonds; and Group 3 samples are blue triangles.

SLC39A6/GATA3				
Dataset	Centroid size	Group 1 p-value	Group 2 p-value	Group 3 p-value
Training dataset	204	0.014245014 (351)	0.009174312 (436)	0.028735632 (522)
Testing dataset	204	0 (274)	0.024154589 (414)	0.001937984 (516)
Sørlie dataset	204	0 (190)	0 (191)	0.004249292 (706)
NKI dataset	124	0 (64)	0.03311258 (151)	0 (247)

Table 2: The p-values for the three groups defined by *SLC39A6* and *GATA3*. The first column lists the datasets in which the three groups were validated at the $\alpha = 0.05$ level. The second column lists the number of genes in the centroids used to classify the dataset to the left. The third through fifth columns list the p-values for each of the three groups.
