# Supplementary material for: Discovery and validation of breast cancer subtypes
Source: BMC Genomics. 2006 Sep 11;7:231. doi: 10.1186/1471-2164-7-231 (PMC1574316; doi:10.1186/1471-2164-7-231)
Supplement: Additional File 3 — This contains the accession information necessary to obtain the microarray data from the Stanford Microarray Database. [file 1471-2164-7-231-S3.pdf]

Table 1: Norway/Stanford dataset

Experiment	Slide name	Experiment	Slide name	Experiment	Slide name
AK-T-001	shds014	AK-T-002	shds061	AK-T-004	shdo042
AK-T-005	shds146	AK-T-006	shds252	AK-T-008	shdo043
AK-T-009	shdo034	AK-T-010	shdo166	AK-T-011	shds149
AK-T-014	shdo168	AK-T-015	shds152	AK-T-016	shds155
AK-T-017	shdo169	AK-T-019	shdo037	AK-T-022	shdo040
ULL-D-007	shcp037	ULL-D-011	shcp052	ULL-D-013	shdm167
ULL-D-016	shdm164	ULL-D-020	shcp044	ULL-D-022	shck150
ULL-D-023	shcp059	ULL-D-026	shcp040	ULL-D-027	shcp049
ULL-D-037	shdm168	ULL-D-038	shdm166	ULL-D-044	shdp127
ULL-D-048	shdm169	ULL-D-053	shdp128	ULL-D-056	shcp075
ULL-D-057	shdm171	ULL-D-065	shco109	ULL-D-066	shcp077
ULL-D-067	shdp129	ULL-D-071	shco110	ULL-D-074	shco111
ULL-D-075	shdp111	ULL-D-080	shcp079	ULL-D-083	shco114
ULL-D-085	shdp130	ULL-D-087	shcp080	ULL-D-096	shco115
ULL-D-099	shco116	ULL-D-101	shdp112	ULL-D-113	shdp114
ULL-D-122	shcp060	ULL-D-132	shdp116	ULL-D-134	shdp234
ULL-D-135	shdp233	ULL-D-139	shdp235	ULL-D-143	shdp117
ULL-D-144	shdp236	ULL-D-150	shdp118	ULL-D-165	shdp119
ULL-D-167	shdp120	ULL-D-169	shcp036	ULL-D-177	shcp050
ULL-D-183	shdp121	ULL-D-184	shdp122	ULL-D-002	shcp033

Table 2: Korean dataset

Experiment	Slide name	Experiment	Slide name	Experiment	Slide name
K-D-003	shcr138	K-D-004	shcr139	K-D-005	shcr140
K-D-006	shcr144	K-D-009	shcr142	K-D-010	shcr131
K-D-011	shcb141	K-D-012	shcb142	K-D-013	shcb143
K-D-015	shcb147	K-D-016	shcb148	K-D-017	shcb149
K-D-018	shcb150	K-D-019	shcb151	K-D-020	shco103
K-D-021	shcb153	K-D-022	shco104	K-D-025Q	shco105
K-D-026	shcb140	K-D-028	shco090	K-D-029	shco091
K-D-030	shco092	K-D-031	shcb161	K-D-032Q	shcb162
K-D-034	shcb163	K-D-037	shco106	K-D-038	shco088
K-D-040	shco093	K-D-041	shco094	K-D-042	shco095
K-D-044	shco097	K-D-047	shco099	K-D-050	shco107
K-D-061	shco123	K-D-069	shco121	K-D-079	shco122
K-D-080	shef186	K-D-111	shef066	K-D-112	shef056
K-D-114	shef070	K-D-114Q	shef091	K-D-115	shef132
K-D-116	shef133	K-D-117	shef134	K-D-118	shef135
K-D-120	shef136	K-D-121	shef072	K-D-122	shef235
K-D-123	shef152	K-D-124	shef046	K-D-125	shef047
K-D-126	shef153	K-D-127	shef155	K-D-128	shef237
K-D-129	shdq186	K-D-130	shef049	K-D-131	shef092
K-D-132	shef156	K-D-134	shdq187	K-D-135	shef050
K-D-136	shef187	K-D-137	shef051	K-D-138	shef007
K-D-142	shef172	K-D-143	shef218	K-D-144	shef087
K-D-145	shef173	K-D-150	shdp239	K-D-151	shdp240
K-R-007	shcr141	K-R-158	shef171	K-Pap-035	shcb164
K-Med-062	shef238	K-Med-096	shef217	K-Med-096Q	shef089
K-Med-097	shef216	K-Med-098	shef090	K-Mu-001	shcr137
K-Pap-051	shco100				

Table 3: Sørlie dataset

Experiment	Slide name	Experiment	Slide name	Experiment	Slide name
BC-A	svcc1114	BC-H2	shac112	BC-HBC3	shac113
BC-HBC4-T1	svn015	BC-HBC5	svl037	BC-HBC6	svn007
BC-FUMI01-BE	shaz112	BC-FUMI02-BE	shby236	BC-FUMI04-BE	shby028
BC-FUMI05-BE	shaz110	BC-FUMI06-BE	shaz104	BC-FUMI07-BE	shaz116
BC-FUMI08-BE	shby033	BC-FUMI09-BE	shby022	BC-FUMI10-BE	shaz140
BC-FUMI11-BE	shby245	BC-FUMI12-BE	hug109	BC-FUMI14-BE	shby041
BC-FUMI15-BE	shaz121	BC-FUMI16-BE	shaz132	BC-FUMI17-AF	shaz124
BC-FUMI18-BE	shby042	BC-FUMI19-BE	shbg128	BC-FUMI20-BE	shby020
BC-FUMI22-BE	shbg110	BC-FUMI23-BE	shaz129	BC-FUMI24-BE	shaz133
BC-FUMI25-BE	shby043	BC-FUMI26-BE	shaz125	BC-FUMI27-BE	shby046
BC-FUMI29-BE	shaz134	BC-FUMI30-AF	shaz135	BC-FUMI35-BE	shaz114
BC-FUMI37-BE	shaz126	BC-FUMI39-BE	shaz131	BC-FUMI40-BE	shby249

Table 3: Sørlie dataset

BC-FUMI41-BE	shby049	BC-FUMI43-BE	shby050	BC-FUMI44-BE	shby021
BC-FUMI45-BE	shby051	BC102B-BE	shby038	BC104A-BE	svl012
BC105A-BE	shby040	BC106B-BE	svl006	BC107B-BE	svcc98
BC108A-BE	svl003	BC11-FA	Sp088	BC110B-BE	svcc78
BC111A-BE	svcc122	BC111B-BE	svcc68	BC112B-BE	svcc53
BC114A-BE	svcc81	BC115B-BE	svcc106	BC116A-BE	svcc108
BC117A-BE	svcc88	BC118B-BE	svcc134	BC119A-BE	svl015
BC120A-BE	svl027	BC121B-BE	svl016	BC123B-BE	svcc89
BC124A-BE	svcc111	NA-BC1257	svcc1077	BC125A-BE	svl018
BC1369	svcc132	BC14	svcc61	BC16	svcc137
BC17	svcc119	BC18	svl034	BC2	svcc96
BC20-FA	svl107	BC201B-BE	shac110	BC205A-BE	shby039
BC206A-BE	svcc93	BC208A-BE	svcc107	BC210B-AF	shac107
BC213B-BE	svcc76	BC214B-BE	svcc92	BC23	svcc99
BC24	svcc100	BC303B-BE	svl020	BC305A-BE	svcc131
BC307B-BE	svl103	BC308B-BE	svcc87	BC309A-BE	svcc51
BC31-0	s104	BC35-0	svcc118	BC37-FA	svcc117
BC38	svcc115	BC4-LN4	svl002	BC40	svl109
BC402B-BE	svcc83	BC404B-BE	svl033	BC405A-BE	svl029
BC406A-2dumb	shac093	BC44	svl108	BC45	svl110
BC46-LN46	svl007	BC48-0	svl022	BC503B-BE	shac091
BC6	svl106	BC601A-BE	shby035	BC605B-BE	svl031
BC606B-AF	svl026	BC608B-BE	svl036	BC610A-BE	shac100
BC702B-BE	svl041	BC703B-BE	shby037	BC704B-AF	svl035
BC706A-BE	svl039	BC708B-BE	svcc104	BC709B-BE	svcc84
BC710A-BE	svcc101	BC711B-BE	svcc120	BC713A-BE	shac092
BC790	svcc130	BC805A-BE	svcc105	BC807A-BE	svcc55
BC808A-BE	svcc124				

Table 4: Zhao/Langerød dataset

**Experiment**	**Slide name**	**Experiment**	**Slide name**	**Experiment**	**Slide name**
BC-D-007	shck182	BC-L-014	shce150	BC-D-015	shbt053
BC-D-017	shck178	BC-D-025	shbt059	BC-D-031	shck163
BC-D-032	shck179	BC-D-035	shcb131	BC-D-038	shcb132
BC-D-041	shcr133	BC-D-045	shck164	BC-D-047	shck177
BC-D-048	shcb129	BC-D-056	shck183	BC-D-057	shcb134
BC-D-060	shbt072	BC-D-063	shcr135	BC-D-065	shcr136
BC-D-070	shck184	BC-D-085	shck181	BC-D-101	shce159
BC-L-075	shbt079	BC-L-042	shcr134	BC-L-058	shce152
BC-L-090	shce162	ULL-D-201	shcp082	ULL-D-002	shcp033
ULL-D-202	shcp073	ULL-D-214	shcp074	ULL-D-216	shcp063
ULL-L-069	shcb155	ULL-L-112	shcb157	ULL-L-014	shcp034
ULL-L-019	shcb145	ULL-L-024	shcp048	ULL-L-031	shcb146
ULL-L-088	shcp081	ULL-L-097	shcp072	ULL-L-105	shcb160
ULL-L-111	shcp066	ULL-L-168	shcp061	ULL-L-181	shcb156
ULL-L-190	shdm163	ULL-L-222	shdm161	ULL-L-028	shcp041
